# Impact of a mid‐urethral synthetic mesh sling on long‐term risk of systemic conditions in women with stress urinary incontinence: a national cohort study

**DOI:** 10.1111/1471-0528.16917

**Published:** 2021-10-05

**Authors:** P Muller, I Gurol‐Urganci, R Thakar, MR Ehrenstein, J Van Der Meulen, S Jha

**Affiliations:** ^1^ London School of Hygiene & Tropical Medicine London UK; ^2^ Royal College of Obstetricians and Gynaecologists London UK; ^3^ Croydon University Hospital Croydon UK; ^4^ University College London London UK; ^5^ British Society of Urogynaecology London UK; ^6^ Sheffield Teaching Hospitals Sheffield UK

**Keywords:** Autoimmune disease, colposuspension, fascial sling, fibromyalgia, incontinence surgery, midurethral synthetic mesh sling insertion, myalgic encephalomyelitis, severe adverse events, stress urinary incontinence, systemic conditions, urogynaecology

## Abstract

**Objective:**

To compare the incidence of systemic conditions between women who had surgical treatment for stress incontinence with mesh and without mesh.

**Design:**

National cohort study.

**Setting:**

English National Health Service.

**Population:**

Women with no previous record of systemic disease who had first‐time urinary incontinence surgery between 1 January 2006 and 31 December 2013, followed up to the earliest of 10 years or 31 March 2019.

**Methods:**

Competing‐risks regression was used to estimate hazard ratios (HR), adjusted for patient characteristics, with HR > 1 indicating increased incidence following mesh surgery.

**Main outcome measures:**

First postoperative admission with a record of autoimmune disease, fibromyalgia or myalgic encephalomyelitis up to 10 years following the first incontinence procedure.

**Results:**

The cohort included 88 947 women who had mesh surgery and 3389 women who had non‐mesh surgery. Both treatment groups were similar with respect to age, socio‐economic deprivation, comorbidity and ethnicity. The 10‐year cumulative incidence of autoimmune disease, fibromyalgia or myalgic encephalomyelitis was 8.1% (95% CI 7.9–8.3%) in the mesh group and 9.0% (95% CI 8.0–10.1%) in the non‐mesh group (adjusted HR 0.89, 95% CI 0.79–1.01; *P* = 0.07). A sensitivity analysis including only autoimmune diseases as an outcome returned a similar result.

**Conclusions:**

These findings do not support claims that synthetic mesh slings cause systemic disease.

**Tweetable abstract:**

No evidence of increased risk of systemic conditions after stress incontinence treatment with a mesh sling.

## Tweetable abstract

No evidence of increased risk of systemic conditions after stress incontinence treatment with a mesh sling.

## Introduction

Stress urinary incontinence is a common condition affecting up to 40% of postmenopausal women that can have a significant effect on quality of life.^1^, ^2^ Conservative treatment options include lifestyle interventions and pelvic floor muscle training.^3^ Further treatments include various surgical options, such as colposuspension, where sutures are used to elevate the proximal urethra, or a mid‐urethral sling insertion.^4^ Slings can be made from a length of fascia harvested from the patient (‘non‐mesh’ sling), or from synthetic material (‘mesh’ sling). Mesh slings were introduced in the 1990s. A randomised controlled trial with a follow‐up period of 6 months, published in 2002, found comparable effectiveness and a similar risk of complications with them compared with colposuspension.^5^ Mesh slings became the dominant surgical treatment for stress urinary incontinence in many countries, with over 200 000 mesh sling insertions performed in the USA in 2010.^6^ In the English National Health Service (NHS), the annual number of mesh sling insertions increased to 16 000 in 2009,^7^ then subsequently decreased, as reports of harmful adverse effects increased.^8^

From 2017, the use of mesh as a treatment of urinary incontinence was restricted or banned in an increasing number of countries.^9^ In 2020 a national review, commissioned by the English Department of Health and Social Care, reported numerous testimonies of women suffering from adverse events, including mesh exposure and systemic conditions, such as autoimmune disease, chronic pain and fatigue.^10^, ^11^ In the USA, there have been successful lawsuits over complications following surgery with mesh for pelvic organ prolapse, and there is media and public concern about the use of mesh in urogynaecological surgery generally.^12^

A number of recent population‐based studies have reported on the long‐term risk of removal and reoperation following mesh sling insertion.^13^, ^14^, ^15^ However, only one study has looked at the incidence of autoimmune disease after vaginal mesh surgery. That study, which was carried out in New York State, compared 1500 women who had mesh surgery for pelvic prolapse with women who had vaginal hysterectomy for benign gynaecological conditions or colonoscopy, and did not find evidence of an association.^16^

The aim of our study was to test the hypothesis that there is no difference in the 10‐year incidence of certain systemic conditions, including autoimmune disease, fibromyalgia and myalgic encephalomyelitis, between women who have urinary incontinence surgery using mesh and those who have urinary incontinence surgery without mesh.

## Methods

We used administrative hospital records to identify all women who had first‐time urinary incontinence surgery in the English NHS between 2006 and 2013, and evaluated the incidence of subsequent admissions with systemic disease within 10 years of the initial surgery.

### Data sources

Data on all inpatient admissions to NHS hospitals in England from April 2002 to March 2019 were extracted from Hospital Episode Statistics, an administrative database with records including patient demographics, dates of admission and discharge, diagnostic and procedure information, and date of death. Procedures for stress urinary incontinence were identified using Office for Population Censuses and Surveys Classification of Interventions and Procedures Version 4 (OPCS‐4) codes (Table S1).^17^

### Cohort selection and outcome definition

All women who had a first‐time urinary continence surgery with or without mesh between 1 April 2006 and 31 March 2013 were eligible for inclusion. The start of the inclusion period was chosen as mesh‐specific OPCS‐4 codes only became available in 2006, and the end was chosen to allow at least 5 years of follow up for each patient. Mesh surgeries evaluated included tension‐free vaginal tape or transobturator tape insertion; non‐mesh surgeries included colposuspension and non‐mesh slings (see Table S1).

Women were excluded if they had a record of previous urinary incontinence surgery in a record of a hospital admission in the 3 years before surgery (mesh or non‐mesh surgery, or use of a bulking agent; see Table S1). We also excluded women who had a record of autoimmune disease, fibromyalgia or myalgic encephalomyelitis in the same period to ensure as much as possible that our outcome reflects the first recording of these conditions.

The outcome was time from urinary continence surgery to the date of the hospital admission with the first recording of a diagnostic code indicating the presence of at least one of 29 autoimmune diseases, fibromyalgia, or myalgic encephalomyelitis, coded according to International Classification of Diseases version 10 (see Table S2).^18^

A woman's ethnicity was retrieved from the record of the admission during which the urinary incontinence surgery took place. If the ethnicity information was not available in that record, but was available in another record, information from that record was used instead. The Indices of Multiple Deprivation are an area‐based deprivation measure and were grouped into quintiles and used to measure socio‐economic deprivation.^19^ The number of pre‐existing comorbid conditions at the point of surgery was generated using the algorithm developed by the Royal College of Surgeons of England to identify conditions that would contribute to a patient's Charlson Score,^20^ applied to records of the admission with the urinary incontinence surgery and all admissions in the three preceding years.

### Statistical methods

Patient characteristics (ethnicity, age, deprivation quintile, number of pre‐existing comorbidities) and year of surgery were described according to type of surgery, using percentages and means.

We estimated the cumulative incidence of systemic conditions, considering death as a competing event. Follow up for each woman ended at the admission during which one of the defined systemic conditions was recorded, at the end of the study period (31 March 2019), or after 10 years of follow up, or death; whichever happened first. A Fine–Gray competing risks regression model was used to test the difference in the cumulative incidence and to estimate the subdistribution hazard ratio (HR) associated with mesh compared with non‐mesh surgery, with adjustment for differences in patient characteristics between the surgery groups in age, ethnicity, socio‐economic deprivation, number of pre‐existing comorbidities and year of operation.^21^ The subdistribution HR can be interpreted as a measure of relative risk: a value of 1 implies no association, a value greater than 1 indicates an increased incidence, and a value below 1 indicates a decreased incidence, with mesh surgery.

Patients with missing data for the patient characteristics included in the regression were excluded when estimating the adjusted results. However, we carried out a sensitivity analysis using multiple imputation with chained equations to deal with the missing ethnicity data.^22^ Model coefficients were obtained from ten imputed data sets, pooled using Rubin’s rules. A second sensitivity analysis was carried out to investigate the impact of restricting the outcome to autoimmune diseases only.

## Results

A total of 95 318 women were identified who had a first‐time urinary incontinence procedure with mesh and 3674 women who had a procedure without mesh between 2006 and 2013. After excluding 6656 women with a systemic condition recorded in their surgery admission or in the three preceding years, 88 947 women who had mesh surgery and 3389 women who had non‐mesh surgery were included.

The total numbers of women having first‐time mesh surgery increased every year until 2008 and then gradually decreased (Table 1; Figure S1). The annual numbers of non‐mesh surgeries fell throughout the inclusion period.

**Table 1:** Characteristics of patients analysed by type of surgery

	Mesh group	Non‐mesh group
	88 996	3432
Follow up (years), median (IQR)	8.7 (6.8–8.7)	9.9 (7.4–9.9)
Year of operation, *n* (%)		
2006	5410 (6.1)	728 (21.3)
2007	11 823 (13.3)	618 (18.1)
2008	13 129 (14.8)	486 (14.2)
2009	12 850 (14.4)	400 (11.7)
2010	12 317 (13.8)	341 (9.9)
2011	11 803 (13.3)	294 (8.6)
2012	11 060 (12.4)	282 (8.2)
2013	10 604 (11.9)	283 (8.1)
Missing	0 (0.0)	0 (0.0)
Age at time of operation (years), average ± SD	53.1 ± 12	52.2 ± 12
Missing, *n* (%)	41 (0.0)	43 (1.3)
Socioeconomic deprivation, national quintiles, *n* (%)		
1 Most deprived	14 590 (16.4)	621 (18.1)
2	16 868 (19.0)	664 (19.4)
3	18 746 (21.1)	683 (19.9)
4	19 316 (21.7)	720 (20.9)
5 Least deprived	19 059 (21.4)	713 (20.8)
Missing	417 (0.5)	31 (0.9)
Number of comorbidities, *n* (%)		
0	70 973 (79.8)	2681 (78.1)
1	15 550 (17.5)	649 (18.9)
2	2070 (2.3)	77 (2.2)
3+	403 (0.5)	25 (0.7)
Missing	0 (0.0)	0 (0.0)
Ethnicity, *n* (%)		
White	82 807 (93.1)	3182 (92.9)
Asian/Asian British	1988 (2.2)	83 (2.3)
Black/Black British	7047 (0.8)	47 (1.3)
Other	1317 (1.5)	54 (1.6)
Missing	2177 (2.5)	66 (1.9)

IQR, interquartile range; SD, standard deviation.

The women who had mesh and non‐mesh surgeries were very similar with respect to age, ethnicity, pre‐existing comorbidities and deprivation (Table 1). The average age at surgery was 53.1 years for women who had a mesh procedure and 52.2 years for women who had a non‐mesh procedure. Of the women who had a mesh procedure, 20.2% had a pre‐existing comorbid condition compared with 21.9% of women who had a non‐mesh procedure. The frequency of missing data was very low, with less than 1% missing data for comorbidities and deprivation, less than 2% for age and less than 3% for ethnicity. Eight women with subsequent systemic disease were excluded from the analyses because date of readmission was missing.

The median follow‐up duration mesh was 8.7 years (interquartile range 6.8–10.0 years) for women who received incontinence surgery with mesh and 9.9 years (interquartile range 7.4–10.0 years) for women who had incontinence surgery without mesh. The reasons for ending follow up were similar between women who had mesh and non‐mesh surgery: 89.0% of women who had a mesh procedure and 88.2% of women who had a non‐mesh procedure exited the study before death or first record of a systemic condition (Table S3). Of the 6294 women who had a first record of a systemic condition, the most common were rheumatoid arthritis (26.0% of women), polymyalgia rheumatica (13.9%) and psoriasis vulgaris (10.1%) (Table S4). Myalgic encephalomyelitis was observed in 4.7% and fibromyalgia in less than 1.0% of these women. There were very small differences in the distribution of the specific conditions between the women who had mesh and non‐mesh procedures.

**Figure 1:**
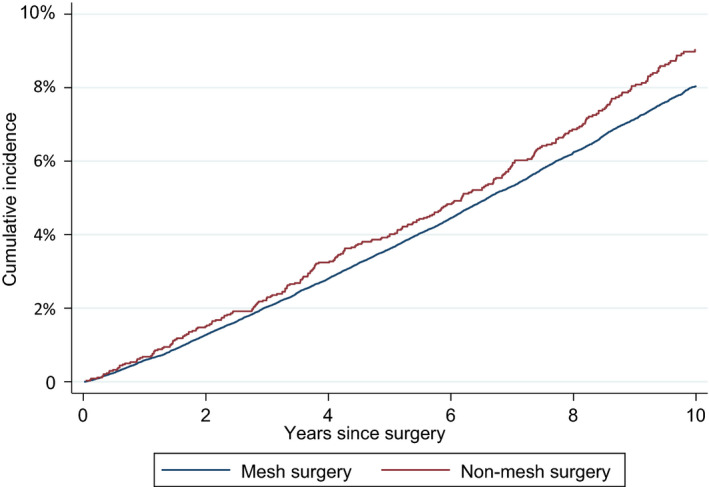
Cumulative incidence of autoimmune disease, fibromyalgia or myalgic encephalomyelitis according to surgery type, with death as competing event.

The cumulative incidence of autoimmune disease, fibromyalgia or myalgic encephalomyelitis was estimated to be 3.6% at 5 years and 8.1% at 10 years in women who had a mesh procedure, and 4.0% and 9.0%, respectively, in those who had a non‐mesh procedure (unadjusted HR 0.89, 95% CI 0.79–1.00, *P* = 0.06; Figure 1 and Table 2).

**Table 2:** Cumulative incidence of autoimmune disease, fibromyalgia or myalgic encephalomyelitis by type of surgery

	Mesh group	Non‐mesh group
Number of patients at risk		
At time of surgery	88 947	3389
At 1 year	88 258	3351
At 5 year	84 110	3192
At 10 year	29 123	1678
Cumulative incidence of systemic diseases, % (95% CI)		
At 1 year	0.6 (0.5–0.6)	0.7 (0.4–1.0)
At 5 year	3.6 (3.5–3.7)	4.0 (3.4–4.7)
At 10 year	8.1 (7.9–8.3)	9.0 (8.0–10.1)
HR (95% CI)	0.89 (0.79–1.01), *P* = 0.06	
Cumulative incidence of autoimmune disease only, % (95% CI)		
At 1 year	0.6 (0.5–0.6)	0.7 (0.4–1.0)
At 5 year	3.4 (3.3–3.6)	3.9 (3.3–4.6)
At 10 year	7.7 (7.5–7.9)	8.7 (7.7–9.7)
HR (95% CI)	0.88 (0.78–0.99), *P* = 0.04	

The estimates of the differences in the cumulative incidence of systemic conditions results were similar when regression modelling was used to adjust for differences in patient characteristics (adjusted HR 0.89, 95% CI 0.78–1.01; Table 3). Older age at first surgery, pre‐existing comorbidities and higher level of socio‐economic deprivation were all strong risk factors for the incidence of systemic conditions, but operation year and ethnic background were not associated with it.

**Table 3:** Hazard ratios expressing the impact of patient characteristics on cumulative incidence of autoimmune disease, fibromyalgia or myalgic encephalomyelitis by type of surgery

	HR	95% CI	*P* value
Surgery type			
Non‐mesh	1		0.07
Mesh	0.89	(0.78–1.01)	
Operation year			
2006	1		0.53
2007	0.92	(0.83–1.03)	
2008	1.02	(0.92–1.13)	
2009	0.99	(0.88–1.10)	
2010	0.98	(0.87–1.09)	
2011	0.99	(0.88–1.11)	
2012	0.97	(0.86–1.10)	
2013	0.94	(0.83–1.07)	
Age group (years)			
18–39	1		<0.01
40–49	1.28	(1.15–1.43)	
50–59	1.59	(1.43–1.78)	
60–69	2.15	(1.92–2.40)	
≥70	2.45	(2.18–2.75)	
Deprivation			
1 Most deprived	1		<0.01
2	0.88	(0.82–0.96)	
3	0.85	(0.79–0.92)	
4	0.80	(0.74–0.86)	
5 Least deprived	0.75	(0.69–0.81)	
Number of comorbidities			
0	1		<0.01
1	1.54	(1.45–1.63)	
2	2.28	(2.03–2.56)	
3	2.59	(2.04–3.28)	
Ethnic group			
White	1		0.41
Asian/Asian British	1.08	(0.92–1.26)	
Black/Black British	0.82	(0.61–1.10)	
Other	1.06	(0.87–1.29)	

In the first sensitivity analysis we repeated the regression modelling with multiple imputation to account for missing ethnicity data. This change had very little effect on the estimate of the difference between the surgery groups (adjusted HR 0.89, 95% CI 0.79–1.00, *P* = 0.06; see Table S5). A second sensitivity analysis, where the outcome was restricted just to autoimmune diseases (excluding fibromyalgia and myalgic encephalomyelitis) also produced near‐identical results compared with the analysis of the full composite outcome (adjusted HR 0.89, 95% CI 0.79–1.00; *P* = 0.04; see Table S6).

## Discussion

### Main findings

We did not find evidence that the use of a synthetic mesh sling in stress urinary incontinence surgery increased the long‐term risk of autoimmune disease, fibromyalgia or myalgic encephalomyelitis, compared with non‐mesh incontinence surgery. The results were very similar when the outcome was restricted to autoimmune disease. Older age at surgery, the presence of pre‐existing comorbidities and a higher level of socio‐economic deprivation, were all independently associated with increased long‐term risk of these diseases.

### Strengths and limitations

Our study used national population‐based data on all urinary incontinence operations carried out in the English NHS between 2006 and 2013. It presents a highly representative population with near‐complete follow up, given that only 3.4% of healthcare expenditure in England covers procedures outside the NHS provided by the private sector.^23^ The large study population allowed comparison of the incidence of specific conditions between women who had mesh or non‐mesh surgery.

The procedure coding available in the English HES data made it possible to distinguish urinary continence procedures that used mesh and those that did not. A unique strength of our study is that we were able to compare two cohorts of women receiving different treatments for the same condition, avoiding any confounding from differences in underlying risk of systemic disease between women treated for different conditions. This is in contrast to the study of the impact of vaginal mesh use in prolapse surgery on autoimmune disease carried out in New York State, which used women who had colonoscopy or hysterectomy as the comparison groups.^16^ We only included women who were reported to have had incontinence surgery. We additionally excluded women if they had a record of autoimmune disease, fibromyalgia or myalgic encephalomyelitis in the 3 years before mesh surgery.

It could be argued that fibromyalgia and myalgic encephalomyelitis should not be considered in epidemiological studies alongside autoimmune disease, because they may be sensitive to a different set of risk factors. However, we included them to ensure that our results are relevant to the concerns of women who had or are considering urinary incontinence surgery. A sensitivity analysis only using autoimmune disease as an outcome produced near‐identical results. Additionally, there were no material differences in the incidence of each of the specific conditions included in the composite outcome between women who had mesh and non‐mesh surgery, confirming that our results are robust to the definition of the outcome.

Our study is restricted to information available in the records of hospital admissions. Our outcome therefore includes conditions severe enough to have been recorded in hospital records. There are no obvious reasons to expect that the incidence of systemic conditions between the two surgery groups would have been different if conditions recorded in primary care or during outpatient visits had been included. However, further research using primary care records and outpatient visits is required to confirm this assumption.

### Comparison with other studies

Our results are in line with other studies in the same area. The prevalence of autoimmune disease in the population is estimated to be around 8%.^24^ Our finding of a 10‐year incidence of 8.1% with mesh surgery and 9.0% without mesh is compatible with these estimates. Our estimates of 5‐year cumulative incidence of 3.6% with mesh surgery and 4.0% with non‐mesh surgery are very similar to the results of the New York State study, comparing outcomes after mesh surgery for pelvic prolapse and vaginal hysterectomy, which reported that around 3% of women had been diagnosed with an autoimmune disease irrespective of the procedure after an average follow up of 6 years.^16^ Mesh is also commonly used in inguinal hernia repair. A study, also carried out in New York State, that compared 30 000 men who had hernia repair with about 80 000 men who had a colonoscopy, found no increased risk of autoimmune disease.^25^

It has been hypothesised that the introduction of synthetic material could lead to an upregulation of inflammatory mediators, which may lead to the development of generalised symptoms.^26^ Another possible pathway is that degradation and absorption of the synthetic material would lead to toxic effects affecting the whole body. Our results do not support these pathways as a mechanism linking the use of synthetic mesh to systemic conditions.

In England, a national review was carried out to investigate how the healthcare system had responded to patients reporting poor outcomes after treatment with pelvic mesh.^10^ One of the review’s key lessons, relevant in the context of our study, was that in all countries where synthetic mid‐urethral mesh slings were used there has been a lack of long‐term monitoring of outcomes, without which it is impossible for regulators, patients and their clinicians to fully understand the harms and benefits of available treatment options. Our study demonstrates that some of these long‐term outcomes can be evaluated promptly using existing administrative hospital data. An expansion of this work including data on primary care consultations and outpatient clinic visits is now a key priority, given that this will allow a more complete evaluation, also including conditions that were not recorded in hospital admissions.

### Interpretation

What does our finding – that the use of mesh does not increase the long‐term risk of systemic conditions – mean for urinary continence surgery? First, a recent population‐based follow‐up study in Scotland comparing mesh and non‐mesh urinary incontinence surgery found lower risk of immediate complications after mesh surgery and a similar risk of further incontinence surgery and later complications up to 5 years.^13^ Taken together with the results from the present study, the evidence is that outcomes of urinary incontinence surgery with and without the use of mesh are very similar.

Second, we found that the long‐term risk of systemic disease was slightly higher in women who had surgical treatment without mesh. However, this difference in risk was not statistically significant and the actual difference in risk was very small (<1% difference in the 10‐year risk), which supports our interpretation that the risks of systemic disease are very similar, irrespective of whether or not mesh was used.

Third, it has been recommended that national patient‐identifiable databases be set up to collect details of the mesh slings at the time of the operation, in combination with long‐term adverse events and patient‐reported outcomes.^10^ However as outlined above, our results in combination with those of other epidemiological studies,^13^, ^15^ suggest that such patient registries should have a wider perspective and include all types of urinary incontinence surgery, irrespective of whether mesh is used.

Fourth, there remain questions about the use of surgery for urinary incontinence itself. Urinary incontinence can be a devastating condition with a severe negative impact on a woman’s quality of life. Surgery provides a further treatment option if non‐surgical treatments have not provided sufficient improvement. Provision of comprehensive information on the long‐term benefits and risks from different treatments, including from patient‐reported outcomes, is needed to allow patients to make fully informed treatment decisions.

### Conclusion

We did not find an increased risk of systemic conditions in women who had urinary incontinence surgery with a synthetic mesh sling. The restrictions in the use of synthetic mesh slings as treatment for urinary incontinence cannot be justified on the basis of concerns related to increased long‐term risks of autoimmune disease, fibromyalgia or myalgic encephalomyelitis. Further comparative evidence from other settings, such as primary care, and on long‐term patient‐reported outcomes, is needed to give women considering surgery a complete picture of the likely outcomes from different treatments.

### Disclosure of interests

None declared. Completed disclosure of interests form available to view online as supporting information.

### Contribution to authorship

The study was conceived by SJ and designed by all the authors. PM performed the statistical analysis with support from IGU. IGU, RT, ME, JvdM and SJ assisted with the interpretation of results. PM and JvnM wrote the manuscript with input and revisions from all other authors. Joint senior authors (SJ and JvdM) made an equal contribution to this study and manuscript. PM attests that all listed authors meet authorship criteria and that no others who meet the criteria have been omitted.

### Details of ethics approval

Not required for the analysis of anonymised routinely collected administrative data.

### Funding

PM and IGU are funded by the Royal College of Obstetricians and Gynaecologists.

### Acknowledgements

The authors would like to thank David Cromwell of the Royal College of Surgeons of England for facilitating access to the Hospital Episodes Statistics database, and Hussein Wahedally of the Royal College of Surgeons of England for preparing a data extract for analysis.

### Disclaimer and role of the funder

The authors are solely responsible for any errors or omissions. The opinions expressed in this article are those of the authors and do not necessarily reflect the position of their affiliations. The funder had no role in the design and conduct of the study; collection, management, analysis and interpretation of the data; preparation, review or approval of the manuscript; and decision to submit the manuscript for publication.

### Transparency statement

PM had full access to all the data in the study, and affirms that the manuscript is an honest, accurate and transparent account of the study being reported; that no important aspects of the study have been omitted; and that there are no discrepancies between the study conducted and the study as originally planned.

## Supporting information


**Figure S1**. Total volumes of stress incontinence surgeries recorded in the Hospital Episodes Statistics database in England, 2002–13.
**Table S1**. OPCS‐4 codes used to identify mesh and non‐mesh surgical treatments for stress urinary incontinence.
**Table S2**. ICD‐10 codes used to identify autoimmune diseases, fibromyalgia and myalgic encephalomyelitis.
**Table S3**. Reasons for censoring by surgery type, women operated on during 2006–13 and followed up to March 2019.
**Table S4**. First recorded autoimmune disease, fibromyalgia or myalgic encephalomyelitis recorded after surgery, by surgery type, 2006–13.
**Table S5**. Multiple imputation‐based estimates of the hazard of autoimmune disease or fibromyalgia or myalgic encephalomyelitis by surgery type up to 10 years follow up, from Fine–Gray subhazards model with adjustment for age, operation year, ethnicity, deprivation and comorbidities.
**Table S6**. Hazard of first record of an autoimmune disease up to 10 years follow up by surgery type, from Fine–Gray subhazards model with adjustment for age, operation year, ethnicity, deprivation and comorbidities.

Supplementary Material

## Data Availability

No additional data are available.
