# Supplementary material for: Impact of a mid‐urethral synthetic mesh sling on long‐term risk of systemic conditions in women with stress urinary incontinence: a national cohort study
Source: BJOG. 2021 Oct 5;129(4):664–70. doi: 10.1111/1471-0528.16917 (PMC9292923; doi:10.1111/1471-0528.16917)
Supplement: Supplementary file 1 — Figure S1. Total volumes of stress incontinence surgeries recorded in the Hospital Episodes Statistics database in England, 2002–13. Table S1. OPCS‐4 codes used to identify mesh and non‐mesh surgical treatments for stress urinary incontinence. Table S2. ICD‐10 codes used to identify autoimmune diseases, fibromyalgia and myalgic encephalomyelitis. Table S3. Reasons for censoring by surgery type, women operated on during 2006–13 and followed up to March 2019. Table S4. First recorded autoimmune disease, fibromyalgia or myalgic encephalomyelitis recorded after surgery, by surgery type, 2006–13. Table S5. Multiple imputation‐based estimates of the hazard of autoimmune disease or fibromyalgia or myalgic encephalomyelitis by surgery type up to 10 years follow up, from Fine–Gray subhazards model with adjustment for age, operation year, ethnicity, deprivation and comorbidities. Table S6. Hazard of first record of an autoimmune disease up to 10 years follow up by surgery type, from Fine–Gray subhazards model with adjustment for age, operation year, ethnicity, deprivation and comorbidities. [file BJO-129-664-s001.docx]

**SUPPLEMENTARY MATERIAL**

**List of legends for Supplementary Tables and Figures**

Figure S1. Total volumes of stress incontinence surgeries recorded in the Hospital Episodes Statistics database in England, 2002-2013.

Table S1. OPCS-4 codes used to identify mesh and non-mesh surgical treatments for stress urinary incontinence

Table S2. ICD-10 codes used to identify autoimmune diseases, fibromyalgia, and myalgic encephalomyelitis.

Table S3. Reasons for censoring by surgery type, women operated on during 2006-2013 and followed up to March 2019

Table S4. First recorded autoimmune disease, fibromyalgia, or myalgic encephalomyelitis recorded after surgery, by surgery type, 2006-2013

Table S5. Multiple imputation-based estimates of the hazard of autoimmune disease or Fibromyalgia or ME by surgery type up to 10 years follow up, from Fine & Gray subhazards model with adjustment for age, operation year, ethnicity, deprivation, and comorbidities

Table S6. Hazard of first record of an autoimmune disease up to 10 years follow up by surgery type, from Fine & Gray subhazards model with adjustment for age, operation year, ethnicity, deprivation, and comorbidities

*Figure S1. Total volumes of stress incontinence surgeries recorded in the Hospital Episodes Statistics database in England, 2002-2013.*

*SUI=stress urinary incontinence.TVT=tension free tape. (open) indicates abdominal surgery whilst (laparo) indicates laparoscopic surgery. Unspecified SUI operations are those with the generic OPCS-4 code M53.8 (“Other specified vaginal operation to support outlet of female bladder”).*

*Table S1. OPCS-4 codes used to identify mesh and non-mesh surgical treatments for stress urinary incontinence*

| **Code** | **Description** | **Surgery group** |
| --- | --- | --- |
| M53.3 | Introduction of tension-free vaginal tape | Mesh |
| M53.6 | Introduction of transobturator tape | Mesh |
| M52.1 | Suprapubic sling operation | Non-mesh |
| M51.1 | Abdominoperineal suspension of urethra | Non-mesh |
| M52.3 | Colposuspension of neck of bladder | Non-mesh |

*Table S2. ICD-10 codes used to identify autoimmune diseases, fibromyalgia, and myalgic encephalomyelitis.*

| **ICD-10 code** | **Disease** | **Group** |
| --- | --- | --- |
| E10 | Type 1 diabetes | Autoimmune |
| M05–M06 | Seropositive rheumatoid arthritis | Autoimmune |
| E05.0 | Thyrotoxicosis | Autoimmune |
| K51 | Ulcerative colitis | Autoimmune |
| M31.5–6, M35.3 | Polymyalgia rheumatica | Autoimmune |
| H20 | Iridocyclitis | Autoimmune |
| G35 | Multiple sclerosis | Autoimmune |
| M45.9 | Ankylosing spondylitis | Autoimmune |
| M35.0 | Sjogren’s syndrome | Autoimmune |
| E06.3 | Autoimmune thyroiditis | Autoimmune |
| D69.3 | Idiopathic thrombocytopenic purpura | Autoimmune |
| M32.1, M32.9 | Systemic lupus erythematosis | Autoimmune |
| D51.0 | Pernicious anemia | Autoimmune |
| K73 | Autoimmune hepatitis | Autoimmune |
| L63 | Alopecia areata | Autoimmune |
| G61.0 | Guillain–Barre syndrome | Autoimmune |
| M34 | Systemic sclerosis | Autoimmune |
| L80.9 | Vitiligo | Autoimmune |
| E27.1 | Primary adrenocortical insufficiency | Autoimmune |
| M31.3 | Wegener’s granulomatosis* | Autoimmune |
| G70.0 | Myasthenia gravis | Autoimmune |
| M33 | Dermatopolymyositis | Autoimmune |
| K74.3 | Primary biliary cirrhosis | Autoimmune |
| L12 | Pemphigoid | Autoimmune |
| D59.1 | Autoimmune hemolytic anemia | Autoimmune |
| L10 | Pemphigus | Autoimmune |
| L40 (-L40.4) | Psoriasis vulgaris | Autoimmune |
| K90.0 | Celiac disease | Autoimmune |
| K50 | Crohn’s disease | Autoimmune |
| G93.3 | Postviral fatigue syndrome (ME) | (Other) |
| M79.9 | Fibromyalgia | (Other) |
| *Renamed to Granulomatosis with polyangiitis (4A44.A1) in ICD-11. | | |

*Table S3. Reasons for censoring by surgery type, women operated on during 2006-2013 and followed up to March 2019*

| **Censoring reason** | **Mesh surgery** | **Non-mesh surgery** |
| --- | --- | --- |
| End of follow up | 79 162 (89.0%) | 2 989 (88.2%) |
| First admission with systemic condition | 6 022 (6.8%) | 272 (8.0%) |
| Death | 3 763 (4.2%) | 128 (4.2%) |

*Table S4. First recorded autoimmune disease, fibromyalgia, or myalgic encephalomyelitis recorded after surgery, by surgery type, 2006-2013*

|  |  | **n (%)** | | |
| --- | --- | --- | --- | --- |
| **ICD-10 codes** | **Disease type** | **Mesh** | **Non-mesh** | **Total** |
| M05, M06 | Seropositive rheumatoid arthritis | 1 558 (26.0%) | 75 (27.7%) | 1 633 (26.0%) |
| M315, M316, M353 | Polymyalgia rheumatica | 837 (14.0%) | 32 (11.8%) | 869 (13.9%) |
| L40 | Psoriasis vulgaris | 608 (10.1%) | 27 (10.0%) | 635 (10.1%) |
| E10 | Type 1 diabetes | 448 (7.5%) | 21 (7.8%) | 469 (7.5%) |
| K51 | Ulcerative colitis | 408 (6.8%) | 18 (6.6%) | 426 (6.8%) |
| K90 | Celiac disease | 325 (5.4%) | 13 (4.8%) | 338 (5.4%) |
| G933 | Myalgic Encephalomyelitis (ME) | 282 (4.7%) | 11 (4.1%) | 293 (4.7%) |
| K50 | Crohn’s disease | 261 (4.4%) | 10 (3.7%) | 271 (4.3%) |
| D510 | Pernicious anemia | 200 (3.3%) | 14 (5.2%) | 214 (3.4%) |
| G35 | Multiple sclerosis | 168 (2.8%) | 7 (2.6%) | 175 (2.8%) |
| E050 | Thyrotoxicosis | 158 (2.6%) | 5 (1.9%) | 163 (2.6%) |
| M350 | Sjogren’s syndrome | 145 (2.4%) | 8 (3.0%) | 153 (2.4%) |
| M321, M329 | Systemic lupus erythematosis | 98 (1.6%) | 10 (3.7%) | 108 (1.7%) |
| E063 | Autoimmune thyroiditis | 68 (1.1%) | 0-4 (<2%) | 68-72 (<1.5%) |
| H20 | Iridocyclitis | 57 (1.0%) | 0-4 (<2%) | 57-61 (<1.5%) |
| D693 | Idiopathic thrombocytopenic purpura | 58 (1.0%) | 0-4 (<2%) | 58-62 (<1.5%) |
| K743 | Primary biliary cirrhosis | 52 (0.9%) | 0-4 (<2%) | 52-56 (<1.5%) |
| G700 | Myasthenia gravis | 48 (0.8%) | 0-4 (<2%) | 48-52 (<1.0%) |
| M799 | Fibromyalgia | 44 (0.7%) | 0-4 (<2%) | 44-48 <1.0%) |
| E271 | Primary adrenocortical insufficiency | 40 (0.7%) | 0-4 (<2%) | 40-44 (<1.0%) |
| M34 | Systemic sclerosis | 32 (0.5%) | 0-4 (<2%) | 32-36 (<1.0% |
| G610 | Guillain–Barre syndrome | 23 (0.4%) | 0-4 (<2%) | 23-27 (<1.0%) |
| K73 | Autoimmune hepatitis | 21 (0.4%) | 0-4 (<2%) | 21-25 (<1.0%) |
| D591 | Autoimmune hemolytic anemia | 16 (0.3%) | 0-4 (<2%) | 16-20 (<1.0%) |
| M33 | Dermatopolymyositis | 13 (0.2%) | 0-4 (<2%) | 13-17 (<1.0%) |
| L12 | Pemphigoid | 11 (0.2%) | 0-4 (<2%) | 11-15 (<1.0%) |
| L63 | Alopecia areata | 11 (0.2%) | 0-4 (<2%) | 11-15 (<1.0%) |
| M313 | Wegener’s granulomatosis | 9 (0.2%) | 0-4 (<2%) | 9-13 (<1.0%) |
| L10 | Pemphigus | 0-4 (<0.1%) | 0-4 (<2%) | 0-8 (<1.0%) |

*Table S5. Multiple imputation-based estimates of the hazard of autoimmune disease or Fibromyalgia or ME by surgery type up to 10 years follow up,* *from Fine & Gray subhazards model with adjustment for age, operation year, ethnicity, deprivation, and comorbidities*

|  | **Subhazard ratio** | **P-value** | **95% Confidence Interval** |
| --- | --- | --- | --- |
| **Operation type** |  |  |  |
| Non-mesh | 1.00 |  |  |
| Mesh | 0.89 | 0.06 | (0.79, 1.00) |
| **Operation year** |  |  |  |
| 2006 | 1.00 |  |  |
| 2007 | 0.96 | 0.42 | (0.87, 1.06) |
| 2008 | 1.02 | 0.74 | (0.92, 1.12) |
| 2009 | 0.99 | 0.87 | (0.89, 1.10) |
| 2010 | 0.98 | 0.74 | (0.88, 1.09) |
| 2011 | 0.99 | 0.90 | (0.89, 1.11) |
| 2012 | 0.97 | 0.67 | (0.87, 1.10) |
| 2013 | 0.95 | 0.43 | (0.84, 1.08) |
| **Age group** |  |  |  |
| 18-39 | 1.00 |  |  |
| 40-49 | 1.26 | <0.01 | (1.14, 1.40) |
| 50-59 | 1.57 | <0.01 | (1.41, 1.74) |
| 60-69 | 2.14 | <0.01 | (1.92, 2.38) |
| >=70 | 2.40 | <0.01 | (2.14, 2.68) |
| **Deprivation** |  |  |  |
| 1 Most deprived | 1.00 |  |  |
| 2 | 0.86 | <0.01 | (0.80, 0.93) |
| 3 | 0.84 | <0.01 | (0.78, 0.90) |
| 4 | 0.79 | <0.01 | (0.73, 0.85) |
| 5 Least deprived | 0.73 | <0.01 | (0.67, 0.79) |
| **RCS Charlson Score** |  |  |  |
| 0 | 1.00 |  |  |
| 1 | 1.54 | <0.01 | (1.46, 1.64) |
| 2 | 2.25 | <0.01 | (2.01, 2.52) |
| 3+ | 2.61 | <0.01 | (2.07, 3.29) |
| **Ethnic group** |  |  |  |
| White | 1.00 |  |  |
| Asian / Asian British | 1.08 | 0.33 | (0.92, 1.26) |
| Black / Black British | 0.81 | 0.15 | (0.60, 1.08) |
| Other | 1.02 | 0.85 | (0.84, 1.24) |

*Table S6. Hazard of first record of an autoimmune disease up to 10 years follow up by surgery type,* *from Fine & Gray subhazards model with adjustment for age, operation year, ethnicity, deprivation, and comorbidities*

|  | **Subhazard ratio** | **P-value** | **95% Confidence Interval** |
| --- | --- | --- | --- |
| **Operation type** |  |  |  |
| Non-mesh | 1.00 |  |  |
| Mesh | 0.89 | 0.04 | (0.79, 1.00) |
| **Operation year** |  |  |  |
| 2006 | 1.00 |  |  |
| 2007 | 0.93 | 0.16 | (0.83, 1.03) |
| 2008 | 1.03 | 0.62 | (0.93, 1.14) |
| 2009 | 0.99 | 0.86 | (0.89, 1.09) |
| 2010 | 0.94 | 0.24 | (0.84, 1.04) |
| 2011 | 0.95 | 0.39 | (0.85, 1.07) |
| 2012 | 0.97 | 0.62 | (0.87, 1.09) |
| 2013 | 0.92 | 0.17 | (0.81, 1.04) |
| **Age group** |  |  |  |
| 18-39 | 1.00 |  |  |
| 40-49 | 1.24 | <0.01 | (1.12, 1.38) |
| 50-59 | 1.61 | <0.01 | (1.45, 1.79) |
| 60-69 | 2.16 | <0.01 | (1.94, 2.39) |
| >=70 | 2.41 | <0.01 | (2.15, 2.69) |
| **Deprivation** |  |  |  |
| 1 Most deprived | 1.00 |  |  |
| 2 | 0.91 | 0.13 | (0.84, 0.98) |
| 3 | 0.87 | <0.01 | (0.81, 0.94) |
| 4 | 0.83 | <0.01 | (0.77, 0.89) |
| 5 Least deprived | 0.78 | <0.01 | (0.72, 0.84) |
| **RCS Charlson Score** |  |  |  |
| 0 | 1.00 |  |  |
| 1 | 1.79 | <0.01 | (1.69, 1.89) |
| 2 | 3.24 | <0.01 | (2.94, 3.57) |
| 3+ | 4.89 | <0.01 | (4.13, 5.78) |
| **Ethnic group** |  |  |  |
| White | 1.00 |  |  |
| Asian / Asian British | 1.16 | 0.05 | (0.99, 1.34) |
| Black / Black British | 0.89 | 0.43 | (0.67, 1.17) |
| Other | 1.01 | 0.89 | (0.83, 1.23) |
